# Bridging Dementia Care in Japan: The Emerging Role of General Medicine Physicians

**DOI:** 10.3390/jcm14217889

**Published:** 2025-11-06

**Authors:** Takao Yamasaki

**Affiliations:** 1 Department of Neurology, Minkodo Minohara Hospital, Fukuoka 811-2402, Japan; yamasaki_dr@apost.plala.or.jp; Tel.: +81-92-947-0040; 2 Department of Research and Development, Kumagai Institute of Health Policy, Fukuoka 816-0812, Japan

**Keywords:** dementia care, general medicine, integrated care, primary care, aging society, Japan, Community-Based Integrated Care System, family medicine, health policy, person-centered care

## Abstract

As global populations age, dementia has become a major public health challenge that warrants sustainable, person-centered, and community-integrated models of care. In Japan, the recent introduction of board-certified general medicine (GM) physicians, trained across both family medicine and hospital general medicine, has created an opportunity to strengthen dementia care through improved continuity and coordination. This narrative review conceptually examines the emerging role of GM physicians within Japan’s Community-Based Integrated Care System and compares this evolving model with dementia care structures in the United States, the United Kingdom, and Canada. By synthesizing policy documents and published literature, this review outlines how GM physicians can serve as integrative actors bridging outpatient and inpatient care, collaborating with dementia specialists, Initial-phase Intensive Support Teams, and Community-based Comprehensive Support Centers to enhance person-centered support throughout the disease trajectory. While empirical outcome data remain limited, this conceptual framework highlights potential contributions of GM physicians to early detection, care transitions, and interdisciplinary collaboration in dementia care. However, challenges persist, including training variability, workforce shortages, and systemic fragmentation. By situating Japan’s experience within an international context, this review provides a conceptual basis for future empirical studies and policy development aimed at strengthening generalist-led dementia care in aging societies.

## 1. Introduction

As the global population ages, the number of people living with dementia has rapidly increased. Alzheimer’s disease is the most common ailment, accounting for the majority of cases worldwide. Dementia causes profound physical, psychological, social, and economic effects, not only on the patients, but also on their caregivers, families, and society [1,2]. Consequently, dementia has emerged as a major global public health issue and an important national policy priority in many countries. In Japan, the 2019 National Framework for Promotion of Dementia Policies emphasized the need for early detection, community-based support, and coordinated multidisciplinary care, setting the stage for a more integrated approach to dementia management [3,4].

Dementia should not be viewed solely as a medical disease; rather, it is a complex condition requiring a holistic approach that considers an individual’s life context, personal relationships, and broader social environment [2]. Thus, dementia care cannot be confined to symptom management or pharmacological treatment alone. It requires comprehensive, person-centered care that respects the individuality, dignity, and life stories of the patient [2].

The Japanese Medical Specialty Board formally designated “general medicine (GM)” as the nineteenth basic medical specialty in 2014. Training under the new board certification program began in 2018 [5,6,7]. Physicians involved in this emerging specialty are responsible for providing comprehensive, cross-cutting care that spans multiple settings and patient conditions. GM in Japan consists of two subspecialties: family medicine (FM) [8] and hospital general medicine (HGM) [9]. FM focuses on community-based primary care, whereas HGM emphasizes leadership, acute care coordination, and governance within a hospital setting [6,7,8,9].

As the system is in its formative stage, the role of GM physicians, including FM and HGM, has not been firmly established, and their professional identities continue to evolve [6,7,10]. This ambiguity extends to dementia care, although GM physicians are increasingly involved; however, there is no standardized, nationwide role for them in dementia diagnosis or management. Despite these limitations, there is growing recognition of their potential to serve as versatile, community-anchored providers, to ensure continuity of care across outpatient and inpatient settings [7].

Unlike organ-specific specialists, GM physicians care for the whole person. They not only address physical symptoms, but also psychological, social, and environmental problems. Thus, they treat individuals, not just diseases [5]. Their approach focuses on longitudinal, relationship-based management that integrates medical, psychological, and social aspects of health [5,6,7]. Moreover, their training includes ambulatory and hospital care, which ideally positions them to provide seamless support throughout the course of chronic conditions, such as dementia [5,6,7,8,9].

However, despite the increasing policy emphasis on community-based integrated care, the specific contribution of GM physicians within Japan’s dementia care pathway remains unclear. Previous studies have described collaborative dementia care models internationally, yet few have examined how Japan’s newly certified GM physicians, trained in both FM and HGM, can function as integrative actors bridging outpatient and inpatient dementia care. This review addresses this conceptual gap by framing GM physicians as emerging coordinators within the Community-Based Integrated Care System (CBICS) [4].

Furthermore, by comparing Japan’s evolving model with those of the United States (US), United Kingdom (UK), and Canada, countries that have long-standing generalist systems but face persistent care fragmentation, this review seeks to identify specific lessons and transferable strategies relevant to Japan’s ongoing health system reforms. By situating this within Japan’s unique institutional and demographic context, this study contributes a novel, Japan-specific perspective to the global discussion on generalist roles in dementia care.

This review focuses on dementia as a representative condition to determine how GM physicians, while still developing their professional identity within Japan’s healthcare system, can uniquely embody the principles of person-centered care and support the evolving community-based integrated care framework.

## 2. Methods

### 2.1. Literature Search Strategy

This review was designated as a narrative review, chosen to conceptually synthesize literature on the evolving role of GM physicians in dementia care, rather than to quantitatively aggregate as in a systematic or scoping review. The narrative approach was appropriate given the limited availability of comparable outcome data and the emerging nature of GM as a specialty in Japan.

This review synthesized conceptual, policy, and empirical literature relevant to the evolving role of GM physicians in dementia care. The review period covered publications from January 2010 and July 2025, encompassing the development of modern dementia care strategies and the institutionalization of integrated, community-centered care models, as reflected in the World Alzheimer Reports 2010 [11] and 2013 [12]. It also includes key domestic milestones in Japan, such as the introduction of the CBICS in 2012 [4] and the board certification of GM physicians in 2018 [5].

English-language, peer-reviewed articles were identified from the PubMed, Scopus, Web of Science, CiNii and J-Stage databases using combinations of the following keywords: “dementia,” “general medicine,” “primary care,” “family medicine,” “hospitalist,” “generalist physician,” “integrated care,” “Japan,” “United States,” “United Kingdom,” “Canada,” “community-based care,” and “early diagnosis.”

Japanese-language materials were also sourced from official websites and government databases, including the Ministry of Health, Labour and Welfare [3,4,13], the Japanese Medical Specialty Board [5], Japan Primary Care Association [8], and Japanese Society of Hospital General Medicine [9]. These sites provide insight into domestic policy frameworks, including the CBICS [4] and GM physician (FM and HGM) training programs [5,8,9].

Inclusion criteria were as follows: (1) publications or policy documents addressing dementia care organization, integration, or the roles of generalist physicians; (2) studies or reports related to Japan, the US, the UK, or Canada; and (3) English- or Japanese-language sources retrievable through academic or official databases.

Exclusion criteria included (1) single case reports; (2) studies focusing solely on pharmacological interventions without relevance to system-level care; and (3) non-peer-reviewed or unofficial publications.

All literature searches were conducted by the author to ensure consistency. Japanese and English materials were reviewed and synthesized separately to account for contextual and linguistic nuances. The findings were then narratively integrated according to thematic relevance, focusing on the organization of dementia care and the positioning of GM physicians within the CBICS framework.

Although the primary review period covered publications from 2010 onward, four pre-2010 works were intentionally retained because of their foundational or historical significance [14,15,16,17]. These earlier sources provide essential conceptual and policy grounding for interpreting post-2010 developments in dementia care systems and GM training.

While a formal quality appraisal (e.g., risk-of-bias assessment) was not conducted due to the narrative nature of this review, credibility and reliability were ensured by restricting inclusion to peer-reviewed journal articles, official government reports, and institutionally published materials. This approach aligns with accepted standards for narrative and integrative reviews, which prioritize conceptual synthesis over quantitative evaluation.

### 2.2. Terminology and Abbreviations

To ensure clarity and consistency, the following terminology is used when referring to primary care providers and generalist physicians in the different healthcare systems:

- US: Primary Care Physicians (PCPs)—generalist physicians responsible for both first-contact and continuous care [18].
- UK: General Practitioners (GPs)—primary care physicians and gatekeepers within the National Health Service [19].
- Canada: Family Physicians (FPs)—generalists who deliver comprehensive primary care across all age groups [20].
- Japan: Kakaritsuke physicians (Kakaritsuke-I)—community-based physicians who provide ongoing care for familiar patients within their residential area. Unlike GPs in the UK, Kakaritsuke physicians are not gatekeepers nor a formally standardized specialty; rather, they represent a functional role emphasizing relational continuity and accessibility within Japan’s free-access healthcare system [21,22].

In this review, “GM physicians” refers to board-certified generalist physicians, who were trained to provide care in outpatient and inpatient settings [5,6,7]. These include:

- FM physicians who are primarily engaged in longitudinal, community-based outpatient care [8].
- HGM physicians who are based in hospital settings manage acute conditions, multimorbidity, and care transitions [9].

## 3. Global Perspectives on Dementia Care in Primary Care

Dementia care systems vary widely worldwide, reflecting differences in healthcare infrastructure, population needs, and policy priorities. To contextualize Japan’s GM model, this section compares three countries with mature primary care infrastructures and national dementia strategies—the US, the UK, and Canada. These countries demonstrate diverse approaches to achieving community-based dementia care integration and offer useful lessons for Japan’s evolving framework.

Healthcare financing also differs: the UK’s National Health Service and Canada’s Medicare are publicly funded, single-payer systems providing largely free care at the point of use [23,24], whereas the US operates a predominantly private, multi-payer system supplemented by Medicare and Medicaid [25]. Such structural variations shape how dementia care is organized, incentivized, and scaled.

### 3.1. The US

In the US, dementia care is managed within the primary care system, in which PCPs are the first point of contact for most patients and their families [26,27]. A national survey conducted by the Alzheimer’s Association indicated that approximately 82% of PCPs perceive themselves as frontline providers for dementia, which highlights their central role in diagnosis and ongoing management [28,29]. In clinical practice, initial assessments are frequently integrated within routine primary care visits, during which brief cognitive screening tools may be utilized. Patients with suspected cognitive impairment may be referred to neurologists, geriatric psychiatrists, or memory clinics for further evaluation [26,29].

Hospital-based dementia care in the US is usually provided by hospitalists [30]. This division of labor, in which PCPs manage outpatient care and hospitalists oversee inpatient care, has created a structural separation between the two sectors. This may hinder the flow of information and contribute to fragmented management over the course of the disease [30].

Although national guidelines emphasize the importance of early and accurate diagnosis, there are several limitations to effective implementation. PCPs often report insufficient dementia-specific training, limited consultation times, and restricted access to specialists, which reduces their ability to diagnose dementia [26,29]. In fact, only 39% of PCPs report feeling “very comfortable” making a diagnosis [28]. In addition, the prevailing reimbursement structures rarely incentivize comprehensive cognitive assessments, which further discourages dementia detection [26,29].

Service availability is also inconsistent based on geography. While urban centers with large academic hospitals have multidisciplinary memory clinics that offer specialized diagnostic and management services [31], rural communities often lack such resources. This contributes to inequities in diagnosis and caregiver support [31]. Federal initiatives, such as the National Plan to Address Alzheimer’s Disease, aim to enhance coordination and expand training opportunities for PCPs; however, implementation has been inconsistent across regions, leaving significant gaps in practice [32].

Critical appraisal: The US model promotes innovation through diverse care delivery mechanisms but faces scalability challenges due to fragmented financing. Its heavy reliance on specialist referral limits accessibility and continuity, particularly in rural and low-income areas. Lessons for Japan include the importance of aligning reimbursement incentives with early detection and strengthening community–hospital linkages. Despite national initiatives, evidence indicates persistent inequities in diagnostic timeliness and caregiver support between urban and rural regions. These disparities suggest that without systemic financial alignment, effectiveness and equity remain constrained—an important cautionary point for Japan’s GM system.

### 3.2. The UK

In the UK, dementia care is provided through the National Health Service, which has established structured diagnostic pathways that begin with GPs [33]. GPs typically conduct a preliminary screening. If dementia is suspected, patients are referred to specialized memory clinics for formal diagnostic evaluation [34,35]. These clinics include multidisciplinary teams, including neurologists, geriatricians, and mental health professionals, which ensures a more comprehensive approach to diagnosis and care planning.

To improve primary care engagement, the UK has implemented national frameworks, such as the Quality and Outcomes Framework, which provide financial incentives for practices to maintain accurate dementia registries and conduct regular patient reviews [36,37]. These measures have led to increases in dementia prevalence, improved follow-up, and a modest reduction in length of hospital stay. In addition, the Quality and Outcomes Framework has indirectly encouraged GP practices to adopt standardized documentation and regular communication with memory clinics, which has gradually improved the transparency of care pathways.

Significant challenges remain. Although early diagnosis rates have improved, coordination between the GPs and hospitals remains a limitation, with delays often occurring from incomplete integration of diagnostic records across care settings [38]. Community-based care is supported by dementia-friendly initiatives that involve social workers, occupational therapists, and community nurses [39]. These programs, combined with local campaigns to improve awareness and reduce stigma [40], are important steps toward building a supportive environment. They also highlight the use of mobilizing nonmedical resources, such as volunteer networks and local charities, to support formal healthcare efforts.

Despite these efforts, ensuring care continuity, particularly during hospital admissions and discharges, remains problematic. Transitional care often depends on region-specific resources, resulting in a considerable variation in patient experiences. Addressing these gaps is considered a priority for the National Dementia Strategy [16], reflecting ongoing concerns regarding the fragmentation of care pathways, despite system-wide reforms.

Critical appraisal: The UK model exemplifies strong structural coordination and incentivization but struggles with intersectoral data integration. While Quality and Outcomes Framework-linked outcomes show modest improvements in patient monitoring, the model’s scalability relies on robust primary care infrastructure—an area Japan continues to develop. Lessons include the value of national incentives tied to outcome metrics and standardized referral pathways. However, despite structural coherence, improvements in caregiver burden and post-diagnostic support remain uneven, especially across deprived areas. This highlights that structural alignment alone does not guarantee equitable or person-centered outcomes, an aspect Japan should consider in GM-led dementia care.

### 3.3. Canada

In Canada, the responsibility for dementia care rests primarily with FPs, who provide outpatient care for the majority of patients [41]. This system has struggled historically from a shortage of specialists and the absence of a unified national dementia strategy. These issues have hindered timely diagnoses and effective care coordination [42]. The adoption of a national dementia strategy in 2019 was a turning point that standardized approaches across provinces and strengthened system capacity [43].

Surveys suggest that while over 86% of FPs provide care for individuals with dementia, only 41% consider themselves adequately prepared to do so, reflecting persistent training gaps and a need for additional educational opportunities [44]. To address this, several models have emerged, including Primary Care Memory Clinics, which focus on interdisciplinary assessment, collaborative management, and the integration of support services [41,44]. Diagnosis typically begins in the primary care setting, with referrals to memory clinics or specialists, such as neurologists or geriatricians, if necessary [45].

Hospital care is usually provided by hospitalists or geriatric teams [46]; however, as in many health systems, coordination between primary and hospital-based services is inconsistent, particularly in rural and remote regions, where access to specialists is limited [15,42,46]. Innovative programs, such as rural memory clinics, have been established to mitigate geographic disparities and ensure equitable access to services [15].

The national strategy, known as A Dementia Strategy for Canada: Together We Aspire, strives for timely diagnosis, caregiver support, and stigma reduction as key priorities [43]. High-quality dementia care has been increasingly linked to interdisciplinary teamwork, integration with community-based services, and strong physician leadership [47,48]. Despite these improvements, achieving seamless transitions across community, hospital, and long-term care settings remains a challenge, and highlights the need for further system-level reforms [15,42].

Critical appraisal: Canada’s model demonstrates effective community integration and rural outreach, but variability across provinces remains. The model’s emphasis on interdisciplinary teamwork and physician leadership offers transferable insights for Japan’s GM system, particularly regarding rural access and collaborative care. Although the 2019 national strategy enhanced coordination and caregiver training, its success depends heavily on local resource allocation, raising questions about long-term scalability. Compared with the UK’s centralized approach, Canada’s flexibility may be more adaptable to Japan’s regional healthcare diversity.

### 3.4. Comparative Insights and Implications for Japan

Across the US, the UK, and Canada, the balance between primary care coordination and specialist input shapes dementia outcomes. Systems with structured incentives (UK) or interdisciplinary models (Canada) demonstrate improved continuity, while those with fragmented financing (US) experience inequity and variability.

Japan’s CBICS offers an institutional framework that could combine these strengths if primary care roles are clearly defined. The emerging role of GM physicians—bridging outpatient and inpatient care—represents a distinctive adaptation toward achieving equitable, seamless dementia care (Table 1). Unlike the GP- or FP-centered systems in the UK and Canada—where community-based management and hospital care remain largely divided—the Japanese GM model uniquely combines both functions within a single specialty. This dual role parallels the coordination goals of integrated care abroad, while offering a context-specific pathway for more seamless continuity within Japan’s CBICS.

Comparative synthesis: From a comparative standpoint, the UK model shows measurable improvements in diagnostic coverage, Canada demonstrates better rural equity through decentralized innovation, and the US highlights the risks of fragmented financing. Evaluating these systems together suggests that Japan’s GM model should prioritize not only integration and coordination but also measurable patient and caregiver outcomes, particularly in under-resourced communities. This multidimensional comparison strengthens the analytical depth and interpretive rigor of this section and aligns with current scholarly call for critical evaluation of effectiveness, scalability, and transferability.

## 4. The Japanese GM Model: Integrating Outpatient and Inpatient Roles in Dementia Care

### 4.1. Current Landscape of Dementia Diagnosis and Care in Japan

Japan’s healthcare system is based on universal health insurance and free access. It allows patients to seek care at any facility regardless of disease severity or referral status [49,50]. Although this system ensures equity, it also fosters specialist-oriented care pathways, which are reinforced by the lack of a gatekeeping system [49]. Modest surcharges for direct hospital visits have had little effect, and cultural preference for specialist credentials as a marker of trust and quality further sustains this tendency [50].

With respect to dementia, Kakaritsuke physicians provide longitudinal care in the community, but have variable training and expertise. Many began as organ-specific specialists and transitioned to community practice without formal education in primary care or dementia [17,51]. Two groups may be distinguished: certified generalists (GM, FM, HGM) [5,8,9] and hybrid subspecialist–generalist physicians [17,51]. Although the former are capable of high-quality primary care, their numbers remain limited [50]. As a result, >70% of general practitioners still refer suspected dementia cases to specialists [14]. Despite national efforts to emphasize the role of primary care, integration remains limited because of the absence of gatekeeping and the lack of a certification system for Kakaritsuke physicians [49].

A dementia diagnosis is primarily made by dementia specialists or organ-based experts, such as neurologists, geriatricians, psychiatrists, and neurosurgeons, often working in memory clinics or in Medical Centers for Dementia established nationwide in 2008 [52,53,54]. Hospital-based dementia care is usually provided by specialists in acute hospitals, where patients with comorbidities, such as pneumonia, are often admitted directly, often through Kakaritsuke referrals [4,53,55]. This structure promotes fragmentation between outpatient and inpatient care.

To address these limitations, Japan established the CBICS, which coordinates medical, long-term care, welfare, housing, and preventive services for dementia care patients [4,55]. Key personnel include Kakaritsuke physicians, dementia specialists, Dementia Support Physicians, Initial-phase Intensive Support Teams, Clinical Support Teams, municipal agencies, and trained community Dementia Supporters, along with acute care hospitals and long-term care providers. Nevertheless, engagement of Kakaritsuke physicians in dementia care remains inconsistent, as only approximately 30% have dementia-specific training [3].

Overall, GM physicians are expected to have a central role. With expertise spanning outpatient and inpatient care and formal dementia training, GM physicians represent an evolved form of Kakaritsuke practice, bridging community and hospital settings to enhance early detection, continuity, and quality of dementia care [5,8,9]. Section 4.2 describes their roles within this emerging framework.

### 4.2. The Role of GM Physicians in the Current System

**Figure 1:**
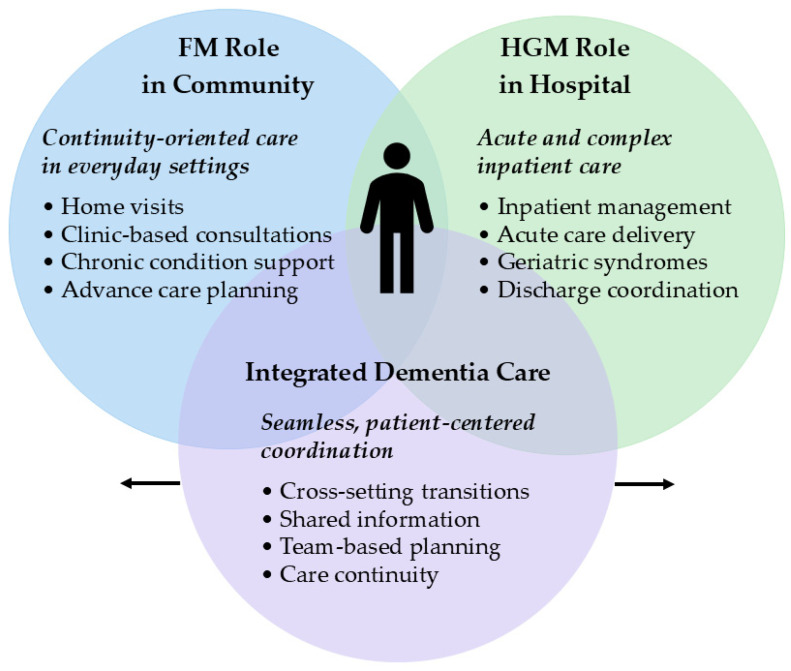
Conceptual framework illustrating the complementary roles of GM physicians in dementia care in Japan. This Venn diagram depicts the two subspecialized domains of GM: FM physicians provide community-based primary care (left), and HGM physicians deliver hospital-based inpatient care (right). The overlapping area represents integrated dementia care, in which GM physicians bridge outpatient and inpatient settings through continuous patient follow-up and coordinated care planning. This integration is expected to yield measurable benefits, such as earlier detection of cognitive decline, reduced avoidable hospitalizations, smooth discharge coordination, and improved continuity of care across the dementia trajectory. The central human icon emphasizes the patient’s position at the heart of this continuum, while bidirectional arrows indicate the dynamic flow of care and information across settings. Abbreviations: FM, family medicine; GM, general medicine; HGM, hospital general medicine.

The complementary functions of FM physicians in community-based primary care and HGM physicians in hospital-based inpatient care, as well as their integration within the GM model, are presented in Figure 1. By combining these competencies, GM physicians can follow patients longitudinally across settings, reducing care fragmentation. As illustrated in Figure 1, this integration is expected to yield measurable benefits such as earlier detection, reduced avoidable hospitalizations, and improved care continuity. A comparative summary of these contributions across primary, hospital, and integrated care settings is listed in Table 2.

- Workforce context: As of 2025, approximately 940 GM physicians are board-certified in Japan, with notable urban concentration and rural shortages reported in national surveys [5]. This uneven distribution underscores the need for strategic workforce planning to ensure equitable service delivery.
- Distinction from Kakaritsuke physicians: Unlike Kakaritsuke physicians, whose community role is functional and uncertified, GM physicians operate under a standardized national curriculum combining FM and HGM competencies. This ensures dual competency in community-based prevention and hospital-based acute management—core to dementia continuity of care.

#### 4.2.1. Coordination Function of GM Physicians

Once GM became a board-certified specialty in 2018 [5], GM physicians, including those trained in FM and HGM [5,8,9], gradually expanded their involvement in dementia care, particularly in regions lacking specialists.

In community settings, FM physicians serve as the primary contact for cognitive concerns [56]. Many utilize standardized tools, such as the Mini-Mental State Examination, Hasegawa Dementia Scale-Revised, or Montreal Cognitive Assessment [51,53]. Although some refer all suspected cases for confirmation, others independently conduct differential diagnoses, rule out treatable causes, and conduct imaging before making a final diagnosis [51]. They also play an important role in advance care planning and preparing families for the disease trajectory [57]. Collaboration with the Initial-phase Intensive Support Team enables timely specialist access for patients without an established Kakaritsuke physician [53].

In hospital settings, HGM physicians manage acute conditions that frequently occur in dementia patients, such as aspiration pneumonia or delirium. They lead inpatient diagnostic reasoning, acute management, and safe discharge planning, which include coordinating multidisciplinary teams to ensure a smooth transition to home or long-term care [58,59,60,61]. This includes addressing cognitive status, caregiver capacity, and available community resources.

Currently, FM and HGM physicians primarily operate within their respective domains, community-based and hospital-based care, which results in fragmented continuity for some patients. This is similar to the occasional disconnect between Kakaritsuke physicians and hospital care during acute illnesses. However, GM physicians, combining FM and HGM competencies, can follow patients across outpatient and inpatient settings, bridging these sectors and reducing care fragmentation. Although full integration remains limited, team-based collaboration within the GM model can approximate seamless care, laying the foundation for an expanded, continuous role [4,10,55].

In the future, the dual capacities of GM physicians will enable them to contribute across the dementia care continuum by detecting early cognitive decline through long-standing patient relationships, managing acute comorbidities in the hospital, and ensuring smooth reintegration into community life. This integrated role not only supports patient-centered care, but also improves capacity for comprehensive dementia management, particularly in under-resourced regions in Japan.

#### 4.2.2. Case Examples of GM-Led Dementia Care

In several prefectures, GM physicians have initiated pilot cross-sector dementia care pathways connecting community clinics, hospital-based specialists, dementia experts, and municipal care managers. These initiatives function as prototypes of scalable CBICS-based models [62,63].

Case 1 (Osaka Prefecture) [62]: A GM physician assumed leadership of a community clinic that had previously relied on part-time specialists and primarily provided chronic disease management. Care continuity was limited, and support for patients with cognitive concerns was minimal. Upon appointment, the GM physician established a multidisciplinary “Dementia Care Team,” launched a memory clinic, and organized regular in-house training for staff. The clinic’s approach emphasized three pillars: (1) sharing diagnostic results and maintaining longitudinal follow-up with patients and families, (2) assessing functional status including Activity of Daily Living and Instrumental Activity of Daily Living and managing comorbidities and Behavioral and Psychological Symptoms of Dementia, and (3) collaborating with care managers to provide comprehensive support for patients and caregivers. Complex cases were referred to the regional Medical Center for Dementia while maintaining ongoing primary care under the GM physician’s supervision. Over time, the clinic gained community recognition, becoming a key node in local dementia care networks, and later expanded into a “Geriatric Care Team” providing comprehensive elderly care. This case highlights the core coordination competency of GM physicians—bridging hospital and clinic care, multidisciplinary teams, community organizations, and families—thereby reinforcing the sustainability of community-based dementia care.

Case 2 (Shiga Prefecture) [63]: In a rural town with limited healthcare resources, most residents previously sought care outside the municipality, and only 18% used a local clinic as their primary care source. After a GM physician, trained in both FM and HGM, was appointed to the clinic, this proportion gradually increased to 30% over the following decade. This shift reflected growing trust in community-based GM and reduced dependence on distant, organ-specific hospitals. The GM physician’s broad competence enabled more comprehensive and continuous primary care within the community, contributing to improved accessibility and coordination across care settings. Although the report did not specifically target dementia or geriatric populations, its implications align with strengthening integrated care frameworks that benefit older adults. However, the report also noted that sustainable implementation requires multi-physician group practice models to prevent overwork and ensure continuous service coverage, aligning with Japan’s ongoing physician workstyle reforms.

Together, these cases demonstrate that GM physicians, integrating FM and HGM competencies, can serve as central coordinators in bridging community and hospital sectors—realizing seamless, sustainable, and patient-centered dementia care.

### 4.3. Integrated Role of GM Physicians: Strengths and Challenges

Japan’s GM-based dementia care model, which integrates the complementary roles of FM physicians and HGM physicians, offers a unique framework for addressing the complex needs of individuals with dementia [51,64]. Traditionally, the continuity of care between Kakaritsuke physicians and hospitals has been fragmented, particularly during acute illnesses. GM physicians are uniquely positioned to bridge this gap by effectively combining community-based primary care and hospital-based inpatient management within a single, integrated role. Thus, GM physicians may be considered an evolved form of Kakaritsuke practice, formalizing and expanding core functions through structured training in FM and HGM. As central players within the CBICS, GM physicians facilitate seamless, person-centered care across outpatient and inpatient settings, ensuring continuity from early detection to end-of-life care [4,55].

**Figure 2:**
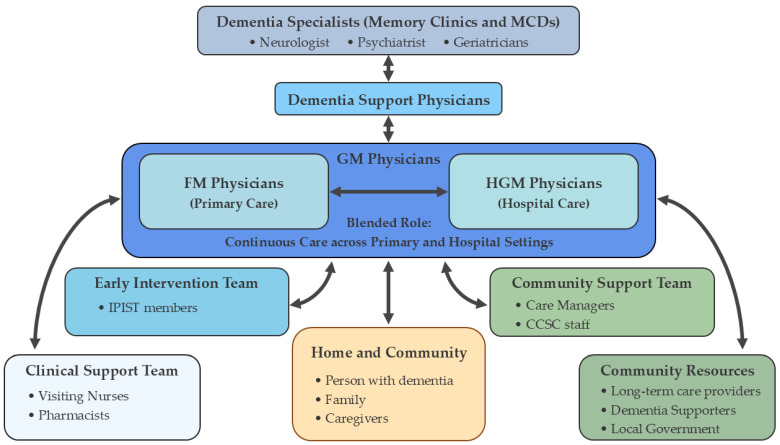
Conceptual framework of the integrated roles of GM Physicians within Japan’s CBICS for dementia care. The diagram illustrates how GM physicians, including FM physicians and HGM physicians, serve as central coordinators linking dementia specialists, dementia support physicians, the early intervention team, the clinical support team, the community support team, and other community resources. Blue hues indicate medical professionals, whereas green represents support staff and community-based resources. Arrows indicate representative care flows, although all stakeholders remain interconnected through continuous collaboration and information sharing. By facilitating bidirectional communication and joint care planning, GM physicians promote person-centered, continuous care spanning the full dementia trajectory from early detection to end-of-life support. This integrated approach is expected to yield measurable improvements, including earlier and more accurate diagnosis, reduced hospital readmissions, smoother care transitions, enhanced caregiver support, and optimized resource utilization within the CBICS network. Abbreviations: CBICS, Community-Based Integrated Care System; CCSC, Community-Based Comprehensive Support Center; FM, family medicine; GM, general medicine; HGM, hospital general medicine; IPIST, Initial-phase Intensive Support Team; MCDs, Medical Centers for Dementia.

Their dual capacities facilitate collaboration with local actors, such as Initial-phase Intensive Support Teams and Community-based Comprehensive Support Centers, thereby integrating medical, social, and long-term care resources [4,55]. Figure 2 illustrates the conceptual framework of the roles of GM physicians across care settings and their interactions with key CBICS stakeholders. This fosters long-term therapeutic relationships, proactive disease management, timely response to complications, and consistent care planning [5,6,7,8,9]. These features are valuable in dementia care, in which evolving cognitive, psychological, and social needs must be holistically addressed [51,52,58,64,65].

Despite these strengths, several challenges remain for the GM-based model. Although GM physicians receive formal training in dementia care, which surpasses that of typical Kakaritsuke physicians, the depth and consistency of hands-on experience and advanced skill development vary, particularly in rural or resource-limited settings where they often serve as frontline providers [10,66]. Personnel numbers do not meet the increasing demand for dementia care, and integration into local care systems remains uneven [67]. Some areas lack the structural support required for collaborative and cross-sectoral roles [49].

Systemic constraints further limit adoption. These include unclear definitions in dementia diagnosis and care coordination, and broader ambiguity regarding the GM physician’s position within the healthcare system [3,5,6,68]. This uncertainty results from the relatively recent formalization of GM as a board-certified specialty [5], and the lack of explicit integration into existing healthcare hierarchies. Other barriers include fragmented care protocols and reimbursement structures that disincentivize cross-sectoral collaboration.

Although Japan has enacted a national dementia strategy, which includes the 2019 National Framework for Promotion of Dementia Policies [13,69] and the 2023 Basic Act on Dementia to Promote an Inclusive Society [13,70], to promote inclusive and evidence-based care, the GM-based model has yet to be formally positioned within international frameworks, such as those advanced by the World Health Organization [71] and the Organization for Economic Cooperation and Development [72]. This lack of explicit alignment may limit the model’s direct transferability; however, it also emphasizes the importance of contextual adaptation and comparative validation. Addressing these challenges will require standardized dementia-specific curricula, strategic workforce planning, role clarification, aligned incentives, and enhanced interprofessional collaboration.

These structural and policy challenges highlight that while Japan’s GM-based dementia care model presents an innovative framework, its integration and sustainability remain uncertain. These issues are further discussed in Section 5, where the model’s transferability, limitations, and future directions are critically examined to inform both policy and practice.

## 5. Discussion, Limitations and Future Directions

Although Japan’s GM-based dementia care model provides a promising framework for enhancing integration between community and hospital settings (Figure 2), its effectiveness remains largely conceptual. The model offers theoretical advantages in promoting continuity, interdisciplinary collaboration, and person-centered care; however, empirical validation is still lacking, and most evidence to date has been descriptive rather than outcome-based [62,63]. Future research should therefore focus on measurable indicators—such as rates of early dementia detection, hospital readmissions, and continuity-of-care outcomes—to evaluate the model’s real-world impact.

Transferability to other healthcare systems requires careful consideration. The GM model is deeply embedded in Japan’s institutional and cultural context, including universal coverage, free access, and the absence of a gatekeeping function [49,50,51]. These contextual differences highlight that the GM model’s success depends not only on clinical competencies but also on system-level alignment between medical, social, and financial structures. In systems with strict referral pathways or different reimbursement incentives (e.g., fee-for-service or capitation models), replicating Japan’s GM-based approach may require structural adaptation. Moreover, the financial mechanisms supporting interdisciplinary and cross-sectoral collaboration are still limited within Japan, suggesting that reimbursement reform is essential before true integration can occur.

Despite the formal board certification of GM in 2018 [5,6,7], GM physicians have not yet been systematically incorporated into Japan’s national dementia strategies, such as the 2019 National Framework for Promotion of Dementia Policies [13,69] and the 2023 Basic Act on Dementia to Promote an Inclusive Society [13,70]. This limited incorporation reflects both the model’s recency and ongoing ambiguity in its role definition relative to existing actors such as Kakaritsuke and Dementia Support Physicians [6,7,10]. Clarifying these relationships and formalizing GM physicians’ responsibilities within national policy frameworks will be crucial for sustainable implementation.

This narrative review has several limitations. First, it relies on conceptual and descriptive synthesis rather than empirical data. The absence of outcome-based evidence limits the ability to evaluate effectiveness, cost-efficiency, or patient and caregiver satisfaction. Second, the GM workforce remains insufficient to meet the rising demand for dementia care, and training pathways vary substantially across institutions, leading to inconsistent competencies. Third, rural–urban disparities persist, as GM physicians are often concentrated in urban or academic centers, leaving peripheral areas under-resourced. Fourth, the model’s recent establishment (since 2018) means that its long-term feasibility, scalability, and policy integration remain uncertain. Finally, while this review compared Japan with the US, UK, and Canada, it did not include other Asian contexts, such as South Korea, China, or Taiwan, where comparable aging trends and community-based care reforms are emerging. Future comparative research in these regions could enhance generalizability and contextual understanding.

Future directions include the empirical validation of the GM model’s outcomes, reform of workforce and training systems, and evaluation of financial incentives that support interprofessional collaboration. Cross-national comparative studies will be essential to determine how Japan’s experience can inform adaptable and culturally sensitive dementia care models in other aging societies.

## 6. Conclusions

Dementia remains a critical public health challenge globally, requiring sustainable and person-centered care models. This narrative review examined how Japan’s newly established GM physicians, trained across FM and HGM, can serve as integrative actors bridging outpatient and inpatient dementia care within the CBICS.

By synthesizing international dementia care models and contextualizing them within Japan’s healthcare system, we developed a conceptual framework highlighting GM physicians as key coordinators. They facilitate early detection, manage care transitions, and align medical and community resources throughout the disease trajectory. This Japan-specific model provides novel insights into the potential of cross-setting generalists to mitigate care fragmentation.

While this review is conceptual, it identifies mechanisms and contextual enablers—including dual training, longitudinal patient relationships, and policy integration—that support GM physicians’ potential impact. Empirical studies are needed to evaluate outcomes such as diagnostic timeliness, care continuity, hospitalization rates, and caregiver satisfaction.

Future research should employ mixed-methods and comparative designs to assess real-world implementation across diverse regions of Japan. Policy efforts are also required to establish standardized training, clear role definitions, and interprofessional collaboration incentives to sustain this integrative model at scale.

Japan’s experience offers valuable lessons for other aging societies confronting similar demographic pressures. By linking conceptual analysis with practical and policy-relevant insights, this review contributes to a coherent, evidence-informed discussion on integrated dementia care models worldwide.

## Figures and Tables

**Table 1:** Comparative Overview of Dementia Care Systems in Japan, the US, the UK, and Canada, with Emphasis on Japan’s Emerging Role of GM Physician Model.

Aspect	Japan	US	UK	Canada
Primary Care Providers	Kakaritsuke physicians; GM physicians (emerging role)	PCPs as the first contact; refer to specialists; hospitalists manage inpatient care	GPs; initial assessment and referral to memory clinics	FPs as first contact; collaborative Primary Care Memory Clinics expanding
Diagnostic Pathways	Specialist-led (memory clinics); limited primary care involvement	Screening by PCPs during routine visits; referral to specialists; fragmented pathways between outpatient and inpatient care	GP-based with structured referral pathways to a memory clinic	Primary care-based, with referrals to specialists or Primary Care Memory Clinics for collaborative assessment
Care Continuity	CBICS provides integrated, continuous care; the GM model may further enhance seamlessness	Often fragmented between outpatient PCPs and inpatient hospitalists, hindering care transitions	Improving, but gaps remain in continuity during hospital admission and discharge	Fragmented, particularly in rural/remote regions with limited specialist access
Community Integration	CBICS; IPIST, dementia support centers involved	Strong integration in academic centers and integrated health systems; limited resources in rural areas	Dementia-friendly communities with strong local government and NHS Trust involvement	Rural memory clinics and local initiatives are expanding to address geographic disparities
Unique Features	GM physicians bridging outpatient and inpatient care; holistic training to support CBICS	PCP-hospitalist division limits continuity; academic centers and integrated systems model team-based dementia care	GP-centered national strategy; structured incentives (Quality and Outcomes Framework); dementia-friendly policies	FP leadership in dementia care; innovative rural memory clinics expanding specialized services

Abbreviations: CBICS, Community-based Integrated Care System; FPs, family physicians; GM, general medicine; GPs, general practitioners; IPIST, Initial-phase Intensive Support Team; NHS, National Health Service; PCPs, primary care physicians.

**Table 2:** Comparative summary of GM physician contributions in dementia care across primary, hospital, and integrated care settings.

Dimension	Primary Care Role (FM Physicians)	Hospital Care Role (HGM Physicians)	Integrated Care Role (Blended GM Role)
Primary Focus and Functions	Early detection of cognitive decline; longitudinal and community-based care	Acute illness management; delirium prevention; discharge planning	Seamless coordination across care settings; person-centered integration
Key Activities and Tools	Cognitive screening (MMSE, HDS-R, MoCA); ACP facilitation; collaboration with IPIST	Symptom control; interdisciplinary teamwork, safe discharge support	Ongoing communication with care managers; facilitation of care transitions
Patient Relationship and Context	Long-term, trust-based relationship in a familiar community context	Short-term but intensive during acute hospitalization	Continuous relationship across disease stages and settings
Systemic Challenges	Limited dementia training; brief consultation time	Discontinuity after discharge; fragmented inpatient-outpatient linkage	Time and system constraints; lack of formal structure for blended roles
Strategic Value within CBICS	Supports early detection and local capacity-building	Ensures safe transitions and acute care support	Embodies CBICS principles; enables holistic, sustainable dementia care

Abbreviations: ACP, advance care planning; CBICS, Community-based Integrated Care System; FM, family medicine; GM, general medicine; HDS-R, Hasegawa Dementia Scale-Revised; HGM, hospital general medicine; IPIST, Initial-phase Intensive Support Team; MMSE, Mini-Mental State Examination; MoCA, Montreal Cognitive Assessment.

## Data Availability

No new data were created or analyzed in this study.
